# Genetic Variations and Differential DNA Methylation to Face Contrasted Climates in Small Ruminants: An Analysis on Traditionally-Managed Sheep and Goats

**DOI:** 10.3389/fgene.2021.745284

**Published:** 2021-09-28

**Authors:** Laure Denoyelle, Pierre de Villemereuil, Frédéric Boyer, Meidhi Khelifi, Clément Gaffet, Florian Alberto, Badr Benjelloun, François Pompanon

**Affiliations:** ^1^ Univ. Grenoble Alpes, Univ. Savoie Mont Blanc, CNRS, LECA, Grenoble, France; ^2^ GenPhySE, Université de Toulouse, INRAE, ENVT, Castanet Tolosan, France; ^3^ Institut de Systématique, Évolution, Biodiversité (ISYEB), École Pratique des Hautes Études | PSL, MNHN, CNRS, SU, UA, Paris, France; ^4^ Institut National de la Recherche Agronomique Maroc (INRA-Maroc), Centre Régional de Beni Mellal, Beni Mellal, Morocco

**Keywords:** adaptation, methylated DNA, whole genome, Morocco, small ruminant, acclimation

## Abstract

The way in which living organisms mobilize a combination of long-term adaptive mechanisms and short-term phenotypic plasticity to face environmental variations is still largely unknown. In the context of climate change, understanding the genetic and epigenetic bases for adaptation and plasticity is a major stake for preserving genomic resources and the resilience capacity of livestock populations. We characterized both epigenetic and genetic variations by contrasting 22 sheep and 21 goats from both sides of a climate gradient, focusing on free-ranging populations from Morocco. We produced for each individual Whole-Genome Sequence at 12X coverage and MeDIP-Seq data, to identify regions under selection and those differentially methylated. For both species, the analysis of genetic differences (F_ST_) along the genome between animals from localities with high vs. low temperature annual variations detected candidate genes under selection in relation to environmental perception (5 genes), immunity (4 genes), reproduction (8 genes) and production (11 genes). Moreover, we found for each species one differentially methylated gene, namely AGPTA4 in goat and SLIT3 in sheep, which were both related, among other functions, to milk production and muscle development. In both sheep and goats, the comparison between genomic regions impacted by genetic and epigenetic variations suggests that climatic variations impacted similar biological pathways but different genes.

## Introduction

The evolution and distribution of species is driven by the variation of their environment. Optimization of the individual’s phenotype to fit the environment may occur on the long-term, as populations adapt to the local values of environmental drivers (i.e., native individuals having on average a higher fitness than migrants, Savolainen et al., 2013). The access to whole genome sequences now allows to study the genomic bases of local adaptation by identifying genes and genomic regions under selection and the environmental parameters responsible for their selection (e.g., honeybees in the Iberian climate Henriques et al., 2018; dogs with the elevation Gou et al., 2014; or goats in the Moroccan climate Benjelloun et al., 2015). In the meantime, the context of global changes lead to an increasing interest on the ability of populations to develop short-term responses to face e.g., climate variations, as well as the interaction between short- and long-term mechanisms. Indeed, populations might quickly react to an environmental change through migration towards more favorable environments, which is well documented in the global warming context (Chen et al., 2011; McDonald et al., 2012). In the short-term, phenotypic plasticity can also increase the ability of organisms to cope with environmental changes. The ability of a genotype to produce, under different conditions, different phenotypes that are best fitted to the environment involves non-genetic mechanisms by which favorable variations can be acquired and even sometimes transmitted. They consist in epigenetic variations, parental effects, ecological and cultural variations (Danchin et al., 2011). One of such mechanism is epigenetics, notably molecular modifications of chromatin without modification of the DNA sequence itself (including e.g., DNA methylation or histone modification) which affects gene expression (Gibney and Nolan, 2010). Until now, most of the studies pointing out the role of molecular epigenetic mechanisms were performed in stress-controlled conditions on plant or animal models. In this case, the effect of one varying parameter on the physiological or epigenetic responses of the organisms is assessed. For example, the glyphosate herbicide injury on *Arabidopsis thaliana* (Kim et al., 2017), or the maternal diet in mice (Cooney et al., 2002) were shown to affect the methylome.

Since the end of the 2000s, the role of these epigenetic processes in ecology and evolution is increasingly being studied (Bossdorf et al., 2008). In this context, epigenetic marks were analyzed in natural populations. For example, the search for epigenetic differentiation by contrasting natural populations of *Lilium bosniacum* living in different habitat conditions (Zoldoš et al., 2018), or in wild baboons (*Papio cynocephalus*) populations with different food resources (natural fodder in a savanna environment or human food scraps, Lea et al., 2016).

Like wild species, farm animals will have to face future climate change, and understanding the genetic and epigenetic mechanisms responding to environmental changes, especially for free-ranging populations, is a key issue for the conservation of Farm Animal Genomic Resources (Bruford et al., 2015). Our study focuses on small domestic ruminants, namely sheep (*Ovis aries*) and goats (*Capra hircus*), which are a very good model for studying local adaptation. Indeed they spread all over the world for about 10,000 years from the domestication center in the Middle East, through human migration and commercial trades (Zeder, 2008). Until recent centuries, they were traditionally managed in a sustainable way leading to populations well adapted to the large variety of environments worldwide (Taberlet et al., 2008). Nowadays, the effect of the environment on farm animals strongly depends on breeding conditions. Intensively farmed animals are rather preserved from environmental variation because they are generally kept indoors with access to quality supply, in contrast to extensive livestock which are raised outdoors with little additional water and feed. Since the rise of the breed concept about 200 years ago, the selection for morphological and productivity traits improved the productivity, but resulted in the loss of genetic variability in many breeds, compromising their ability to adapt to future environmental changes (Taberlet et al., 2008). Thus, in the context of climate change gathering “epigenomic information to be integrated with phenotypic and genomic data to scrutinize the biological basis for adaptation and plasticity/resilience in livestock populations” is a key question in farm animal genomics research (Bruford et al., 2015). Until now, epigenetic analyses on livestock have been performed almost exclusively in stress-controlled conditions in relation with agronomic traits such as reproduction (Lan et al., 2013), production (Peñagaricano et al., 2014; X. Wang et al., 2015) and disease sensibility (Doherty et al., 2014). To our knowledge, only the study by Sevane et al. (2018) searched for DNA methylation in relation with climate variation by comparing cattle adapted to tropical and temperate climates.

In this context we aimed at characterizing both epigenetic and genetic variations with regards to climate variation in a single framework. Our study focused on sheep and goats sampled in Morocco where farming is mainly carried out in traditional ways with a strong impact of environmental conditions upon animals. Within each species, our goal was to identify candidate genomic regions involved in both genetic (selection on Single Nucleotide Polymorphism, SNP) and epigenetic (differential DNA methylation) mechanisms related to environmental variations, using whole genome comparisons of groups of individuals from locations with contrasted annual temperature range.

## Materials and Methods

### Sampling

**FIGURE 1:**
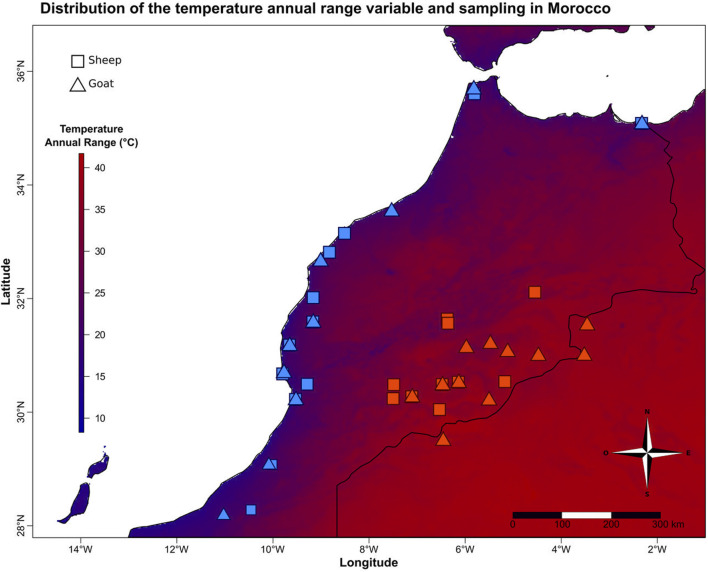
Variation of the annual temperature range and sampling of small ruminants in Morocco. Distribution of sheep and goats sampled at each end of the temperature gradient in Morocco. The color gradient corresponds to the variation of the annual temperature range (BIO7).

Morocco has very contrasted climates including hot/cold desert and semi-arid, warm-summer Mediterranean, dry-summer subarctic (Born et al., 2008). In this context, we sampled sheep (*Ovis aries*) and goats (*Capra hircus*) from locations with contrasted annual temperature variations (Figure 1, bioclimatic variable BIO7 from the WorldClim dataset, www.worldclim.org/bioclim, Hijmans et al., 2005). This variable is the difference between the maximum temperature of the warmest month and the minimum temperature of the coldest month. It is correlated with several temperature variables, sunshine and elevation (Supplementary Table S1). Twenty-one unrelated goats (10 and 11 from low and high annual temperature ranges, respectively) and 22 unrelated sheep (12 and 10 from low and high annual temperature ranges, respectively) were selected (Figure 1; Supplementary Table S2) from the samples collected between January 2008 and March 2012 in the frame of the NextGen European project (Grant Agreement no. 244356), in accordance with ethical regulations of the European Union Directive 86/609/EEC, and for which Whole Genome Sequences were already available (projects.ensembl.org/nextgen/). The average annual temperature range between the two groups is 20 vs. 39.5°C, which is almost twice for the animals living in Eastern Morocco.

### Whole Genome Sequences

#### Dataset

We extracted the states of SNPs from the variant files generated by the NextGen project. Whole Genome Sequences (WGS) at 12–14 X coverage corresponding to the studied individuals were retrieved from the European Nucleotide Archive (www.ebi.ac.uk/ena, accession code PRJEB7436). The sequences, produced and filtered following the protocol in Alberto et al. (2018), were aligned on the reference genomes CHIR v1.0 (GenBank assembly GCA_000317765.147, Dong et al., 2013) for goats and OAR v3.1 (GenBank assembly GCA_000298735.1, Jiang et al., 2014), for sheep.

#### Detection of Selection Signatures

We kept SNPs with a missing data rate inferior to 30% and a minor allele frequency above 1%. Then we calculated the F_ST_ value (Weir and Cockerham, 1984) between the two groups (low vs. high temperature annual range for each species) for each SNP using the weir-fst-pop function in vcftools version 0.1.16 (Danecek et al., 2011). Candidate genomic regions were defined by 1) merging SNPs with the highest F_ST_ values (i.e., top 0.025% and top 0.001% for the pathway and gene approaches, respectively) closer than 5,000 bases from each other and then 2) adding 2,500 bases at each side of the region obtained. At the end 3) the genes overlapping these regions were extracted.

To annotate the genomic regions, two gene ontology analyses were performed with the GO Ontology database released on 2020-08-10 (Carbon and Mungall, 2018). The *Bos taurus* reference was used to annotate the candidate genes. We do not expect any major issue due to the use of *B. taurus* as a reference as the genome synteny between these three Bovidae species is very high (see Jiang et al., 2014).

1) Enrichment analysis: it was performed on the list of genes extracted with the 0.025% threshold. For that, we used the statistical over-representation test in PANTHER (version 15.0, Mi et al., 2019) and the *Bos taurus* reference. Each biological process with a significant Fisher’s exact test and a False Discovery Rate (FDR) lower than 0.05 was annotated. We kept the more basal significant biological pathway and searched the GO terms with the highest hierarchical level (i.e., child term of “biological process”) associated.
2) Candidate gene identification: we extracted the GO term corresponding to the top genes using a more stringent threshold (0.001%) to avoid the false positive and highlight the most impacted genes. In addition, a specific gene bibliography was done using Google Scholar with the gene’s name and “goat/sheep” or “livestock” or “mammals” as keywords, to determine what could be their effect on livestock phenotypes. These were grouped into large phenotype categories such as “Environmental perception,” “Immunity,” “Production” (genes involved in milk or meat production), “Reproduction” and “Other.”

### DNA Methylated Regions

#### DNA Extraction, Library Preparation and Sequencing

DNA was extracted from ear biopsies collected by the Nextgen consortium (see above) using the isolation of genomic DNA from tissues protocol from QIAamp® DNA micro kit (QIAgen, Germantown, MD, United States). Methylated DNA immunoprecipitation sequencing (MeDIP-Seq) was performed at the GeT-PlaGe core facility, INRAE Toulouse (http://www.get.genotoul.fr). Methylated DNA libraries were prepared according to Bioo Scientific’s protocol using the Bioo Scientific NEXTflex™ Methyl-seq Library Prep Kit for Illumina Sequencing. Briefly, DNA was fragmented by sonication on a covaris M220, size selection was performed using AMPure XP beads and adapters were ligated to be sequenced. 3 µg of each library was diluted in 450 µl of TE buffer, denatured in boiling water for 10 min and immediately cooled in ice for 10 min. Then 50 µL of 10X concentrated IP buffer was added to the mixture as well as 1 µg of anti-5-methylcytosine monoclonal antibody (clone 33D3, Diagenode®). After overnight incubation at 4°C with agitation, the DNA-antibody complexes were purified using 40 µL of “Dynabeads-ProteinG” previously washed in PBS. The DNA-antibody complexes were washed twice in 700ul of IP buffer and then the DNA was purified using the iPure kit (Diagenode®). Control or input DNA samples were not coupled to the antibodies and were therefore not enriched in epigenetic markers. Then, 12 cycles of PCR were performed. Library quality was assessed using an Advanced Analytical Fragment Analyzer and libraries were quantified by QPCR using the Kapa Library Quantification Kit. MeDIP-Seq was performed on an Illumina® HiSeq 2500 with the Illumina Reagent Kits. At the end, the sequenced reads correspond to fragment of methylated DNA.

#### Dataset Preparation

Illumina paired-end reads (mean size of 125 bp) from all animals were aligned on the same reference genomes as WGS data by using the default parameters of BWA-MEM version 0.7.12 (Li and Durbin, 2009). The function multicov from bedtools version 2.29.2 (Quinlan and Hall, 2010) was used to report the MeDIP-Seq coverage of alignment files inside windows of 100 non-overlapping base pairs along the genome. The same function was used to extract the WGS coverage. In these windows we also extracted the number of CpG from the reference genomes by using the MEDIPS package (Lienhard et al., 2014) on *R* software version 3.5.3 (R Core Team, 2019).

We compared genome wide coverage profiles of MeDIP-Seq by using the MEDIPS.correlation function from the MEDIPS package with the uniq, extent, and shift parameters equal to 0 and the window size parameter equal to 100, to keep all MeDIP-Seq reads in each 100 bases window on the genome. We verified with a pairwise Pearson test that the number of read by windows for one animal had a correlation coefficient greater than 0.6 with all other individuals of the same species.

The numbers of WGS and MeDIP-Seq reads in each window were normalized between individuals with default parameters of calcNormFactors function (edgeR package, Robinson et al., 2010). Then, normalized MeDIP-Seq read counts were divided by normalized WGS read counts in order to get rid of the effect of sequencing coverage variations (e.g., due to repeated regions). Normalized values have been rounded to two decimals. Four outlying windows (2 for each species) with 10 times more MeDIP-Seq reads than the others were removed as this may be due to PCR or alignment errors.

#### Whole Epigenome Correlations

For each species, correlations between the number of CpG and that of MeDIP-Seq reads found in the 100 bp window were tested using a Pearson’s correlation test. We used the same test to test the correlation between species for each of these two variables. For that we used the correspondence between *Capra* and *Ovis* orthologous regions resulting from the cross-alignment between the two reference genomes CHIR_1.0 and OAR v3.1 performed by Alberto et al. (2018). Results were represented as density plots performed with the hexbin package (Carr et al., 2019).

#### Detection of Differentially Methylated Regions

We used the edgeR package to detect DMRs. Starting from non-standardized MeDIP-Seq read counts, we kept the 100 bp windows for which at least 10% of the samples had at least 1 count-per-million (1 read per million windows, this accommodates differences in library sizes between sample) and we normalized the read counts with the calcNormFactors function with default parameters. We estimated common dispersion with the estimateGLMRobustDisp function which mitigates the influence of outliers (X. Zhou et al., 2014) and we tested the occurrence of differential methylation between groups with the default parameters of the exactTest function. The *p*-values obtained were converted into q-values with the Bioconductor package qvalue (Storey et al., 2015) and the ones showing q-values inferior to 0.01 were selected for the rest of the analysis. Finally, we merged significant windows distant than less than 5,000 bases into regions.

In order to delineate the peaks of methylation occurring for some of the individuals within these regions, we used the changepoint package (Killick and Eckley, 2014) which detects breakpoints in time series. We looked for all possible breakpoints within a region using the cpt.meanvar function that identify change in mean and variance of the sum of coverages for all animals of each group. We kept the region between two breakpoints that contained at least one window detected as significant with edgeR, thus, the delimited regions were merged with the bedr package (Waggott et al., 2017) when they overlapped. Then, we used a Wilcoxon test to detect peaks with different methylation levels between the two groups. Among those peaks, we discarded those where the number of MeDIP-Seq reads was related to the DNA coverage in the WGS data, because here high MeDIP-Seq counts would result from the high DNA coverage (e.g., due to repeated sequences). The regions containing the remaining peaks were mapped on the reference genomes and included or overlapping genes were identified. We used the Functional classification viewed in gene list analysis in PANTHER to extract the Biological Pathways annotated in the Gene Ontology Database associated to these genes.

## Results

### Detection of Selection Signatures

The F_ST_ values at each genome position and their overall distribution are presented for goats and sheep in Supplementary Figures S1, S2, respectively. Almost 94,5% of the F_ST_ values are less or equal to 0.1 for both species. The top 0.025 and 0.001% thresholds correspond to F_ST_ value of 0.4 and 0.55 respectively.

#### Enrichment Analysis

The 5,000 SNPs for goat and 6,000 SNPs for sheep which harbored the highest 0.025% F_ST_ values (Supplementary Figures S3, S4) corresponded to 442 and 489 genes, respectively, putatively under selection.

**FIGURE 2:**
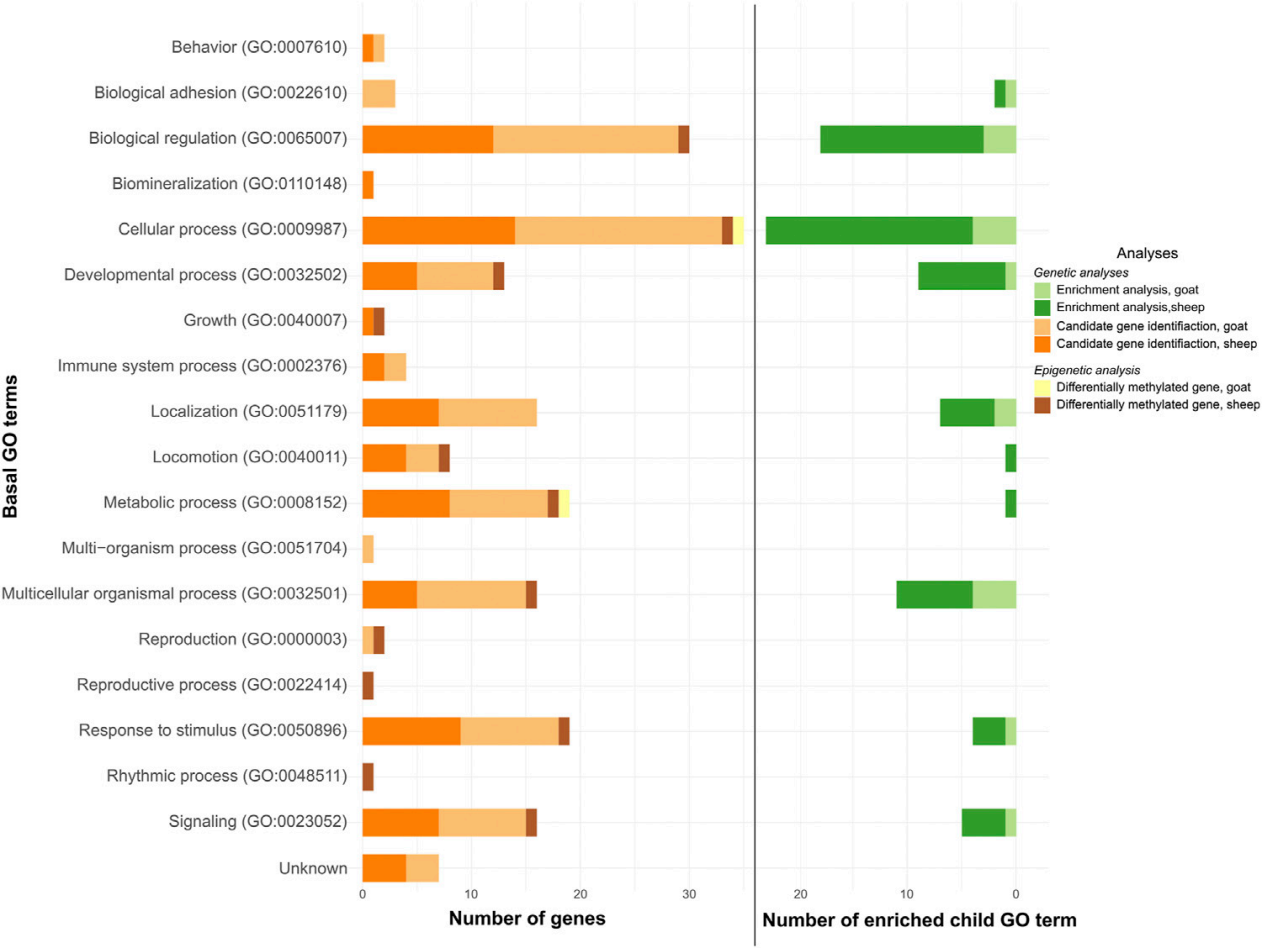
GO terms putatively involved in the adaptation and acclimation of small ruminants to temperature variations. Number of genes or child GO terms per basal GO terms for candidate gene identification (under selection -oranges- and differentially methylated -yellow/brown-) and enrichment analysis (greens), respectively.

The enriched pathways were associated with 8 and 10 GO terms in goat (Figure 2, light green color; Supplementary Table S3) and sheep (Figure 2, dark green color; Supplementary Table S4), respectively. Eight were common to both species.

Among the 0.025% top genes (i.e., 931 genes selected for the enrichment analysis), 41 were orthologous between sheep and goats. When we did the same enrichment analysis (described above) with the 41 genes shared by sheep and goats we obtained 21 GO terms. Four of them had the “Nervous system development” in their parental terms; and the last three were associated with cellular processes, one of which was related to synapses organization.

#### Candidate Gene Identification

The top 0.001% F_ST_ values corresponded to 194 SNPs for goat and 146 SNPs for sheep distributed in 56 and 42 genomic regions, respectively. Their location in the species genome is detailed in Supplementary Tables S5, S6 for goat and sheep, respectively. In goats, 34 of the 56 regions were intergenic and the others were related to 22 genes (Table 1). For sheep, we obtained 24 intergenic regions and 18 genes (Table 1), which differed between the two environmental groups. No genes were common between the two species.

**TABLE 1:** Phenotype categories in which the candidate genes (top 0.001%) are involved.

Species	Phenotype categories	Genes
Goat	Environmental perception	TMTC2, EDIL3, SASH1
	Immunity	—
	Production	CP, SLC9A9, PPFIA2, TMTC2, GALNTL6, CTSB, SASH1, PLCG2, MYADM
	Reproduction	ADGRB3, USH1C, PLCG2, MYADM, SERPINB7
	Other	TRABD2B, MVB12B, KLF12, TNFSF9, CALHM3, SORCS1, RAB30
	Unknown	LOC102184299, LOC106503718
Sheep	Environmental perception	NOX3, KSR2
	Immunity	SAMD12, SEMA5A, SEZ6L
	Production	BMPER, KSR2, PTPRE
	Reproduction	GCSAML, KSR2, BMPER
	Other	AGO3, IQCJ, KCNG3, ANO6, CASP8AP2, CNKSR3, ENGASE, KCNA4
	Unknown	LOC101107868, LOC101119001

The 22 genes were linked to 15 GO terms in goat (Figure 2, light orange color; Supplementary Table S5) and the 18 genes in sheep were associated with 14 GO terms (Figure 2, dark orange color; Supplementary Table S6). Twelve of these GO terms were common to both species.

In livestock, several of these genes are known to be involved in phenotypic changes in relation with the perception of the environment (3 genes in goat, 2 in sheep), immunity (1 in goat, 3 in sheep), production (9 in goat, 2 in sheep), reproduction (5 in goat and 3 in sheep). The “Other” category brings together the genes whose effect on the phenotype was not documented in livestock species. We also detected genes that were uncharacterized (Table 1, Supplementary Tables S5, S6).

### Distribution of CpG and Methylated Regions in Both Species

The majority of the 22 million windows (i.e., 96% for each species) carried one methylated read, 3 and 2% more than one, and 1 and 2% none, for goat and sheep, respectively. Concerning the number of CpG, 47% of the windows did not have any, 50% had between 1 and 4, and 3% had more than 4 in both species.

**FIGURE 3:**
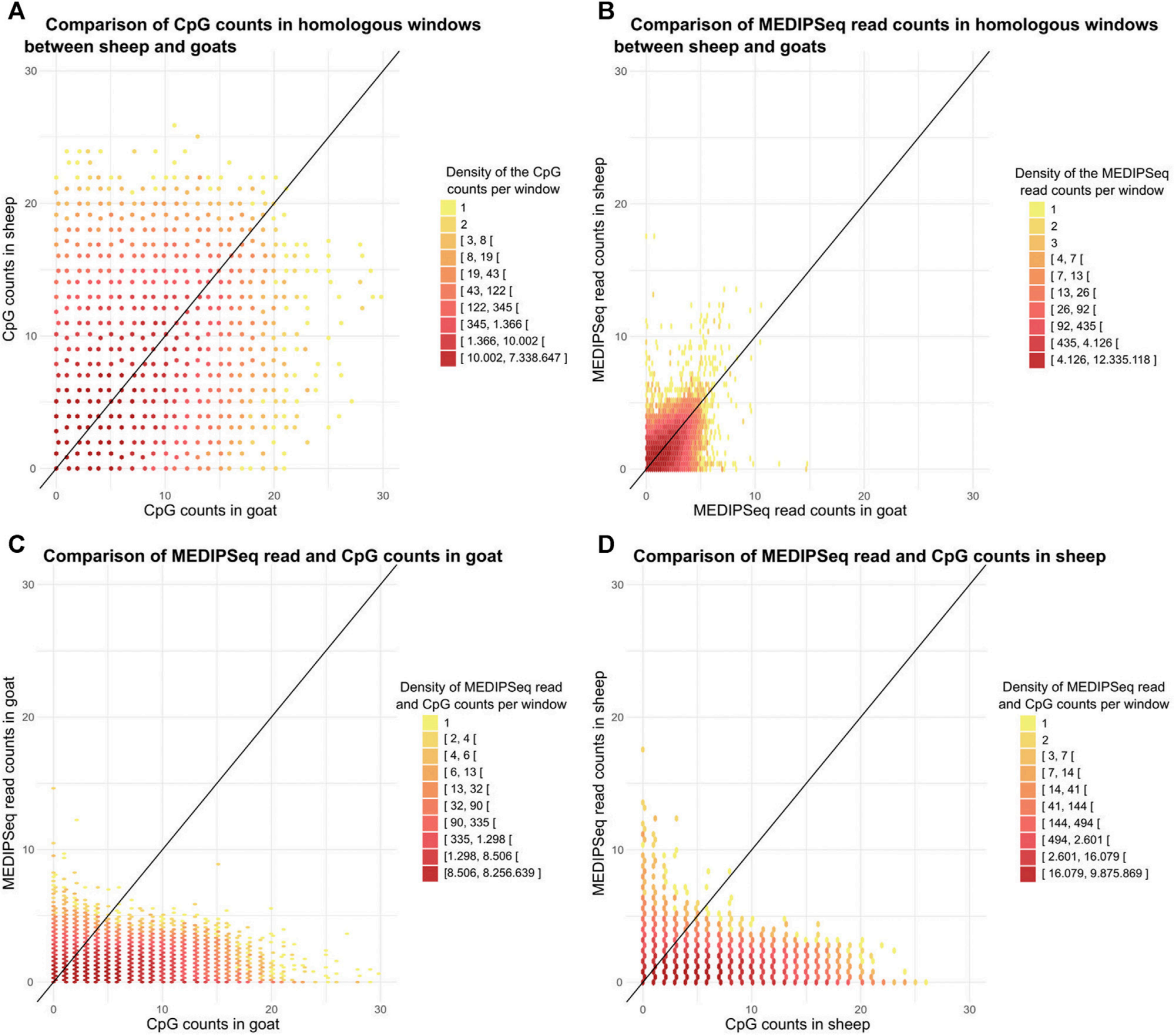
Characterization and comparison of sheep and goats methylomes. Relationship between the number of **(A)**. CpG, or **(B)**. MeDIP-Seq reads per 100 bp window between the two species across the genome. Relation between the number of MeDIP-Seq reads and the number of CpG per 100 bp widow for **(C)**. goat, and **(D)**. sheep. The colors represent the number of points and the black line (y = x) is represented.

Genomic windows of 100 bp that were orthologous between *Capra* and *Ovis* showed a correlation between species for the number of methylated read (R = 0.81, *p*-value < 2.10^-16^, Figure 3A) and the number of CpGs (R = 0.67, *p*-value < 2.10^-16^, Figure 3B) A large number of windows had few CpG and methylated reads (25% carried less than 3 CpGs and 1 methylated read). Within species, we found a correlation between the number of CpGs and that of methylated reads, R = 0.49 (*p*-value < 2.10^-16^, Figure 3C) for goats and R = 0.44 (*p*-value < 2.10^-16^, Figure 3D) for sheep.

**FIGURE 4:**
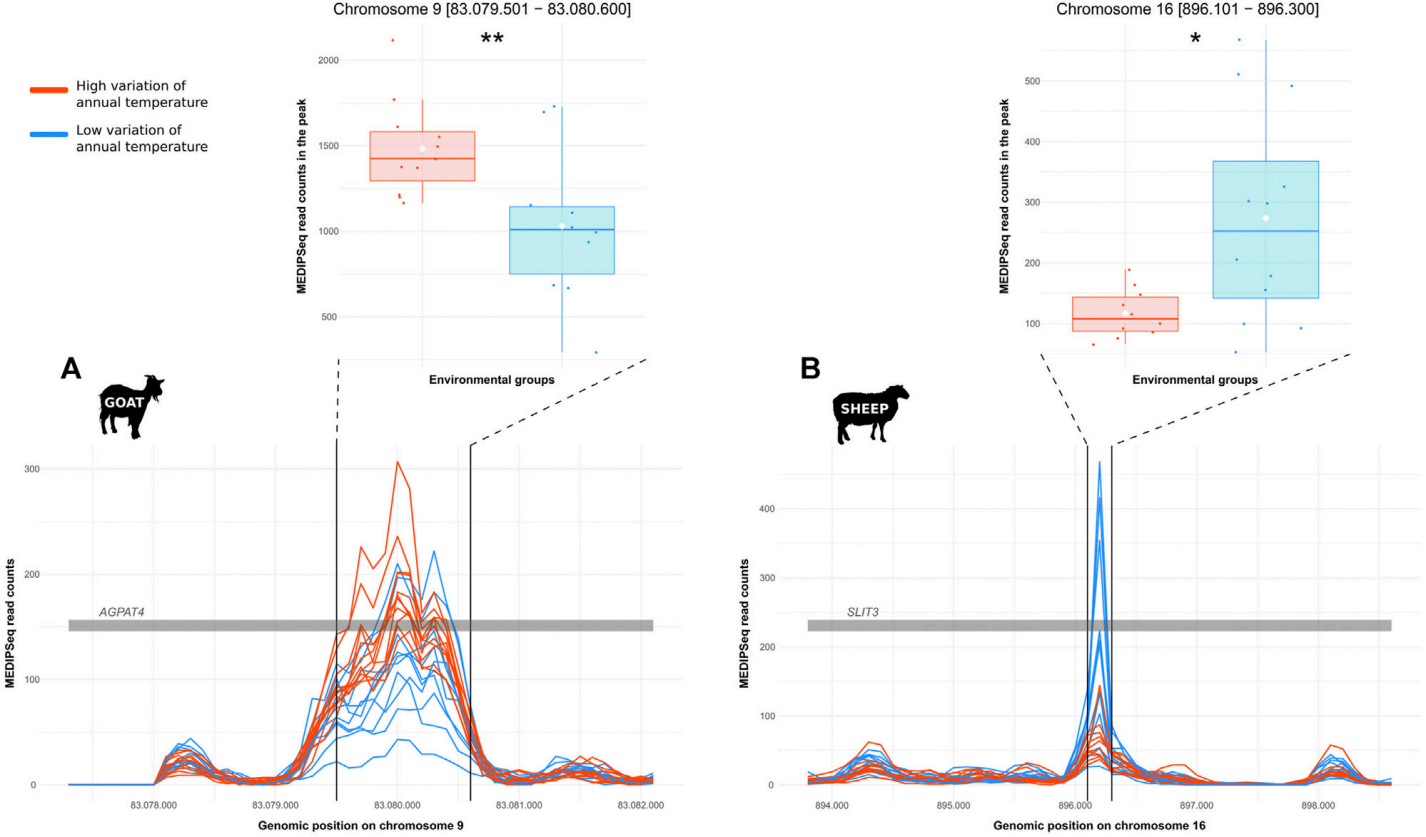
Differentially methylated regions putatively involved in temperature acclimation in small ruminants. DMRs between genomes from locations with low (blue) and high (red) temperature annual range for **(A)**. goat or **(B)**. sheep. Each line represents the variations of the level of DNA methylation along a gene for one individual; the grey bar represents part of the gene; the two black lines delineate the peak on which the differential level of methylation was tested. Boxplots represent the level of methylation (MeDIP-Seq coverage) for each group compared with a Wilcoxon test (Significance codes: ** = *p*-value ≤ 0.01, * = *p*-value ≤ 0.05).

For the detection of DMRs, 20,256 windows in goat and 16,281 windows in sheep were kept out of more than 24 millions. The edgeR analysis revealed 20 and 7 windows with significant differences in methylation levels between the two groups (i.e., low vs high annual temperature variations) in goat and sheep, respectively. Merging candidate windows closer than 5,000 bp, we obtained 8 and 4 regions in which we detected 8 and 5 methylation peaks for goat and sheep, respectively. From these, 4 and 2 peaks respectively showed significant differential MeDIP-Seq coverage between the 2 groups (low vs. high temperature annual range). For 4 peaks, the differential MeDIP-Seq coverage was concomitant to a differential WGS coverage, indicating that the differentiation would probably result from repetition polymorphism. Then only one region per species was kept as resulting from a differential methylation. They were associated to AGPTA4 in goat and SLIT3 in sheep (Figure 4).

The GO terms associated with these genes were “Cellular process,” and “Metabolic process” for AGPAT4 (Figure 2, yellow color); and “Biological regulation,” “Cellular process,” “Developmental process,” “Growth,” “Locomotion,” “Metabolic process,” “Multicellular organismal process,” “Reproduction,” “Reproductive process,” “Response to stimulus,” “Rhythmic process,” and “Signaling” for SLIT3 (Figure 2, brown color).

## Discussion

To our knowledge, this is the first study to investigate simultaneously the genetic and epigenetic variations putatively involved in the adjustment of phenotypes to field conditions in small ruminants. Until now, few publications jointly addressed both mechanisms. Some studies highlighted significant association between local genetic variation and the presence of DMRs like that of Eichten et al. (2013) who found that 51% of the DMRs between maize populations were associated with local SNPs. Foust et al. (2016) and Herrera and Bazaga (2010) found a correlation between AFLP and MS-AFLP marks in two salt marsh perennials (*Spartina alterniflora* and *Borrichia frutescens*) and violet populations (*Viola cazorlensis*), respectively. In our study, we investigated both genetic and epigenetic variations in sheep and goats originating from field populations. The sampling was carried out in order to avoid a breed effect as much as possible. However, given the specific distribution and adaptation of some breeds, several Ghazalia goats and D’man sheep were part of the groups submitted to high temperature annual range (c.a. 39.5°C) as opposed to that with low temperature annual range (c.a. 20°C). They represented less than half of the individuals (3/11 and 4/10 in goats and sheep, respectively) and had similar methylation and F_ST_ patterns to the other breeds from the same group. Thus, the mechanisms inferred would be shared by the variety of Moroccan breeds represented in our sampling. Our study points out several genes which polymorphism or methylation level is contrasted between the two groups and may contribute to the adaptation and acclimatization to these specific environments. The candidate genes putatively under selection are primarily involved in environmental perception, immunity, production and reproduction, and their variations could be driven by different environmental factors. First, a direct effect of high temperatures might operate through thermoregulation by reducing body temperature, through the decrease of metabolic heat and the increase of heat dissipation. This might be obtained by avoiding solar radiations (Al-Tamimi, 2007), increasing respiration or sweating and reducing food intake (Marai et al., 2007; Sejian et al., 2010). These adjustments might reduce the energy allocated to other biological processes including productivity (Tao et al., 2011; Dado-Senn et al., 2019) or reproduction (Meyerhoeffer et al., 1985; Monteiro et al., 2016; Krishnan et al., 2017). In relation with temperature annual range, we found candidate genes involved in production, including milk production for CP, MYAMD, PTPRE, SASH1 and SLC9A9 (Hussein and Staufenbiel, 2012; Dong et al., 2015; Laodim et al., 2017; Shin et al., 2017; Xiang et al., 2017), mammary gland development for PPFIA2 (Mota et al., 2020) and morphology for TMTC2 (Abo-Ismail et al., 2017; Z.-H. Fang and Pausch, 2019). In addition, CTSB, GALNTL6, and KSR2 genes were related to meat production (Russo et al., 2002; Doran et al., 2014; Puig-Oliveras et al., 2014; Lukić et al., 2020), and BMPER and KSR2 with body size (Puig-Oliveras et al., 2014; Zhao et al., 2015; Lukić et al., 2020). Genes involved in reproduction were also impacted. ADGRB3, GCSAML, and KSR2 affect fertility (Pimentel et al., 2011; X. Wang et al., 2015; Mohammadi et al., 2020), while PLCG2 and SERPINB7 affect gestation (Vallée et al., 2003; Ponsuksili et al., 2012). Moreover offspring survival can also be impacted through litter size via BMPER and USH1C (Kwon et al., 2016; Bolormaa et al., 2017; Mota et al., 2020) and offspring weight with MYADM (Gonzalez et al., 2020). Also, we found two candidate genes related to environmental perception. NOX3 is involved in the response to stresses in general (Lambeth, 2004) and has been related with heat stress in chicken (Aggrey et al., 2018), and KSR2 plays a role in the energy balance by adapting feeding behavior and thermogenesis in mice (Guo et al., 2017). Second, environmental parameters covarying with the annual temperature range (BIO7) may be prominent in driving the selection on the genes which polymorphism was contrasted between the two groups. TMTC2 and EDIL3 are involved in adaptation to high altitude in sheep and cattle (Ji Yang et al., 2016; Pierce et al., 2020), this later variable being correlated with BIO7 in Morocco. In addition, BIO7 variable is correlated with sunshine which impacts directly skin pigmentation, which would explain the detection of SASH1, which regulates melanocytes migration in relation with the production and transfer of melanin to protect skin from UV radiations (D. Zhou et al., 2013). Third, the temperature regime may have indirect impacts on phenotypes by driving other environmental factors. Especially, it determines plant resources (i.e., food quality and quantity) and is related to water availability, and thus strongly impacts the diet of free ranging livestock. The lack of resources has a negative impact on, e.g., reproduction (Martin et al., 2004; J. J. Robinson et al., 2006) and lactation (Razzaghi et al., 2016). In contrast, supplementation, which has a positive effect on milk yield and composition in sheep, could temperate this negative impact on lactation (Caroprese et al., 2011). As a candidate gene, we found PLCG2 which is regulated in the cattle liver according to feed intake (Salleh et al., 2017). Likewise, genes involved in immune functions may be selected by multi-factorial drivers related to nutrition or the pathogenic context in relation with climate (Bett et al., 2017). This could be the case for SAMD12 playing a role in pancreatitis in human (Giri et al., 2016), SEZ6L associated with foot-and-mouth disease (Lee et al., 2015) or SEMA5A with mastitis in cattle (Sugimoto et al., 2006).

Besides the candidate genes putatively involved in local adaptation through allelic selection, we identified two strong candidates in relation with an epigenetic response. The global epigenomic context was similar in sheep and goats, and the level of DNA methylation along the genome was correlated to the CpG content. This is consistent with what is known about mammalian epigenomes where the CpG methylation generally covers the entire nuclear genome, with the exception of CpG rich regions near the promoters of active genes (Suzuki and Bird, 2008; Feng et al., 2010). We found two genes differentially methylated according to the provenance of the individual, namely AGPAT4 (1-acylglycerol-3-phosphate O-acyltransferase 4) in goats, and SLIT3 (Slit Guidance Ligand 3) in sheep. AGPAT4 is part of the 1-acyl-sn-glycerol 3-phosphate acyltransferases family which is involved in triglyceride synthesis (Lu et al., 2005; Takeuchi and Reue, 2009), with a role in the milk composition of ruminants (Bionaz and Loor, 2008; He et al., 2011; Jie Yang et al., 2016). It has an organ-related level of expression in human (Lu et al., 2005; Prasad et al., 2011) and plays a role in muscle development and meat quality in cattle (X. Fang et al., 2017). In addition, the variation of AGPAT genes expression in the mammary gland has been related to milk fat depression in dairy cows (Peterson et al., 2003). On its side, SLIT3 takes place into the SLIT-ROBO interactions which are involved in the development of the nervous system (K. H. Wang et al., 1999; Andrews et al., 2007), cardiac development (Liu et al., 2003; Mommersteeg et al., 2015) and reproduction, especially menstrual cycle (Dickinson and Duncan, 2010) and fertility (Amundson et al., 2015). SLIT3 was also shown to be differentially methylated and upregulated in goat mammary glands during lactation compared to the dry period (Zhang et al., 2017). Such DMRs related to milk quality and lactation might result directly from heat stress. Indeed, it was shown that heifers submitted to a thermic shock during fetal development had smaller mammary gland alveoli, resulting in lower milk production (Skibiel et al., 2018; Dahl et al., 2019). The DMRs observed could also result from indirect effects of the climate through a variation of the diet resulting from a shift in plant resources. Peterson et al. (2003) showed that the concentrate forage diet influences the mRNA abundance of AGPAT genes in mammary glands in dairy cows, involving a modification of milk fat. The regulation of the SLIT-ROBO pathway is also affected by the diet (Amundson et al., 2015). Indeed, the diet can directly influence the levels of DNA methylation (Anderson et al., 2012) either due to direct exposure of the individuals or to parental effects. For example, a heat-stress-induced perturbation of mammary glands development in the fetus, related to differential methylation and differential gene expression (Skibiel et al., 2018), results in effects persisting over the lactations and in the next generation too (Ouellet et al., 2020). Maternal nutrition also affects DNA methylation and gene expression of lambs, especially in muscle and adipose tissues (Lan et al., 2013; Peñagaricano et al., 2014; Namous et al., 2018). Obviously, we found differential methylation in genes related to lactation in ear biopsies, not mammary glands. However, sweat glands localized in the dermis and mammary glands have a common tissue origin, and recent studies showed that the most similar tissues had comparable CpG methylation patterns (Lokk et al., 2014). Given the epigenetic memory, embryonic patterns may be retained in adult tissues (Hon et al., 2013). In this case, an early stress (e.g., *in utero*) could modify the methylation pattern of embryonic cells, which could be retained in structures with a common origin. Further characterization of the methylation patterns in different organs, including mammary glands, is needed to confirm the possible role of these mechanisms.

We found that candidate genes for local adaptation in response to different temperature regimes were related to a large variety of biological pathways which were mainly involved in functions such as immunity, perception of the environment and reproduction, or related to productivity traits. While these functions were affected in both sheep and goats, the best candidate genes (i.e., top 0.001%) identified were different, confirming that alternative genes might be selected under the same environmental constraints for close species (e.g., Benjelloun, 2015; Raeymaekers et al., 2017) or even populations of the same species (Manceau et al., 2010; Benjelloun et al., 2015). However, out of the 0.025% top genes (i.e., 931 genes selected for the enrichment analyses), 41 were orthologous between sheep and goats and mainly related to the nervous system development. Interestingly, the present study also investigated epigenetic variations in the individuals displaying candidate genes for local adaptation. Our deliberately stringent approach to avoid false positive, which identified only one candidate DMR per species, prevents a global view but already points out similarities between traits impacted by genetic and epigenetic mechanisms. In sheep, genes impacted by selection such as BMPER, GCSAML, KSR2, PTPRE affect embryo quality, litter size and lactation, which are also influenced by SLIT3 that was differentially methylated. Likewise, in goats, AGPAT4 that was differentially methylated has an effect on lipid metabolism, meat quality and the presence of lipids in milk, these traits being influenced by several candidate genes such as CTSB, MYADM, SERPINB7, USH1C, GALNTL6 and CALHM3.

## Conclusion

This study showed differentially methylated genes and candidate genes under selection related to a differential regime of temperatures underwent by sheep and goats. Genetic and epigenetic mechanisms share several target functions including lactation and milk composition. These adjustments of phenotypes to the two contrasted environments may result from drivers related to temperature either directly or indirectly through e.g., the variation of plant resources conditioning the diet. We do not yet have enough evidence to say that these results reflect the joint effects of selection and epigenetic regulation on the same traits. However, such combined effects might play an important role in evolution (Richards, 2011), and we can wonder whether DMRs might cushion phenotypic variations due to prior selection of genes or reflect the persistence of adaptive plasticity as long as adaptation is not complete? Deciphering the relative impact of climate and e.g., differential diets on the genetic and epigenetically-induced variations of phenotypes will be a major stake for developing future breeding strategies in the context of global change.

## Data Availability

The genetic dataset (fastq of Whole Genome Sequencing) analyzed in this study can be found in the European Nucleotide Archive (www.ebi.ac.uk/ena, accession code PRJEB7436). The epigenetic dataset (fastq of the MeDIP-seq) generated for this study can be found in the European Nucleotide Archive (www.ebi.ac.uk/ena, accession code PRJEB47531).
