# Supplementary figures and images for: Genetic Variations and Differential DNA Methylation to Face Contrasted Climates in Small Ruminants: An Analysis on Traditionally-Managed Sheep and Goats
Source: Front Genet. 2021 Sep 28;12:745284. doi: 10.3389/fgene.2021.745284 (PMC8508783; doi:10.3389/fgene.2021.745284)

# $F_{ST}$ for Moroccan sheep

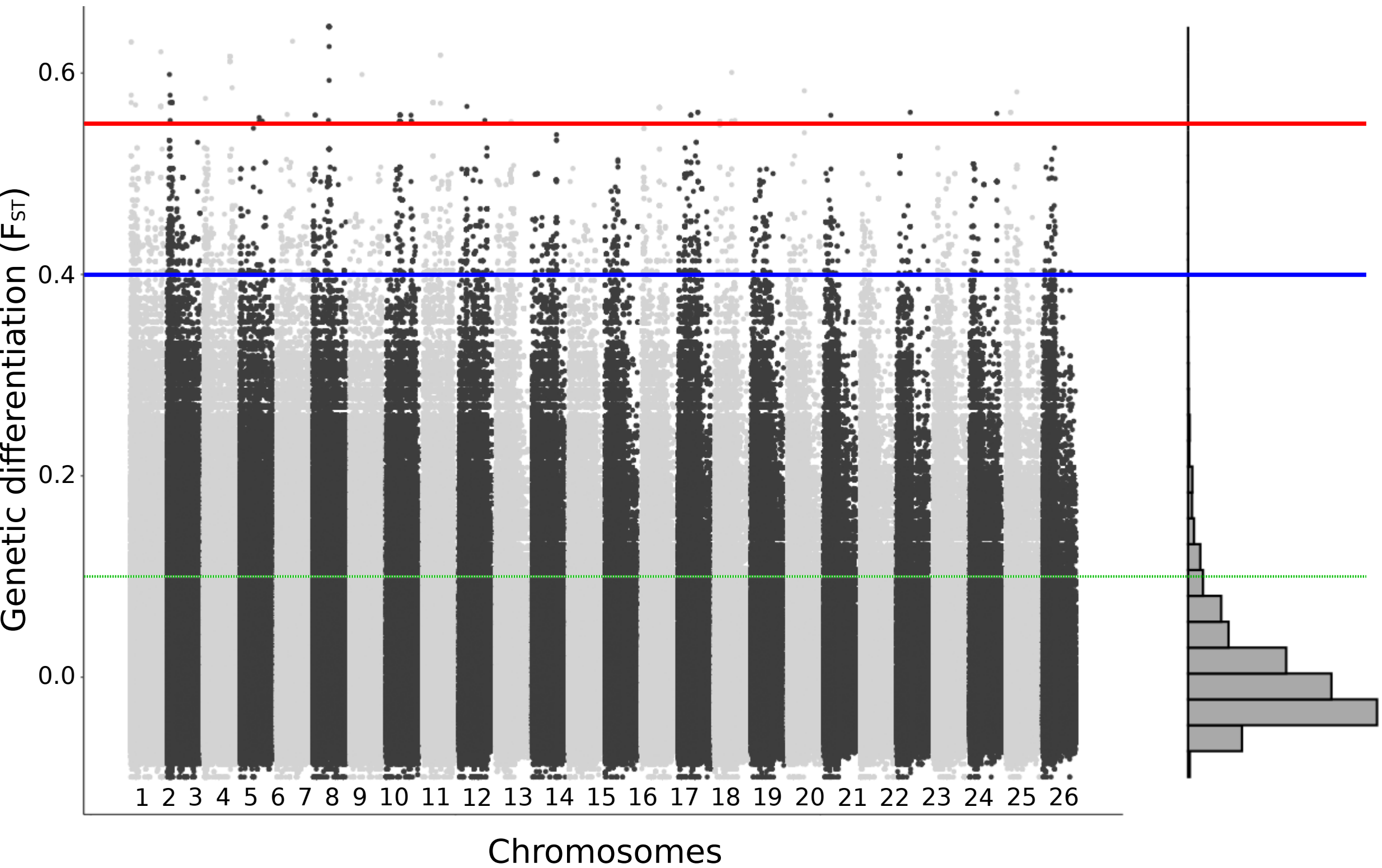

Supplement: Supplementary file 1 [file DataSheet2.PDF]

# $F_{ST}$ for Moroccan goats

Genetic differentiation ( $F_{ST}$ )

0.75  
0.5  
0.25  
0.0

1 2 3 4 5 6 7 8 9 10 11 12 13 14 15 16 17 18 19 20 21 22 23 24 25 26 27 28 29

Chromosomes

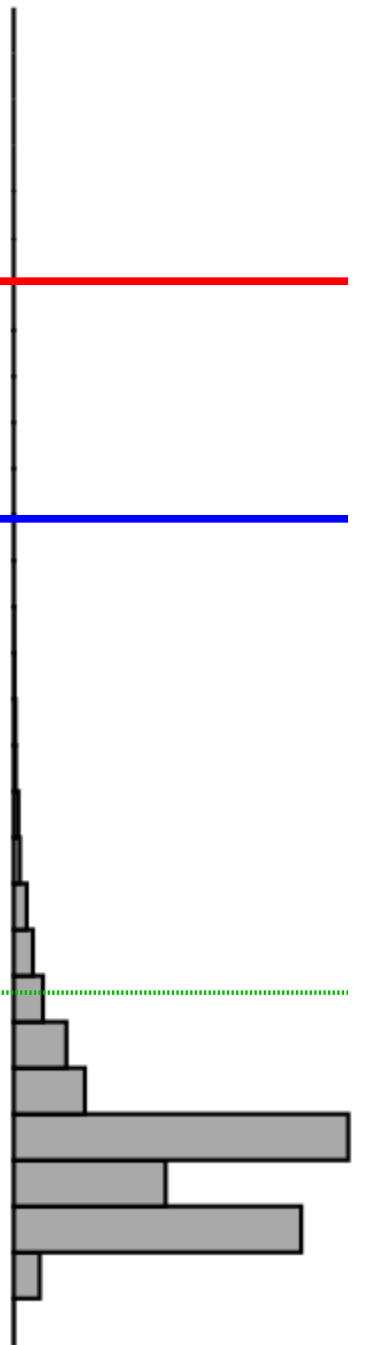

Supplement: Supplementary file 5 [file DataSheet1.PDF]
